# Supplementary material for: The High Content of Ent-11α-hydroxy-15-oxo-kaur- 16-en-19-oic Acid in Adenostemma lavenia (L.) O. Kuntze Leaf Extract: With Preliminary in Vivo Assays
Source: Foods. 2020 Jan 9;9(1):73. doi: 10.3390/foods9010073 (PMC7022889; doi:10.3390/foods9010073)

Supplementary Figure 1

The whole plant powder of *A. lavenia* (named as Ma Zhi Hu) is sold in United States under the permission of medical doctors (the products can be obtained from QualiHerb [https://store.qualiherb.com/products/ma-zhi-hu]).

QualiHerb
13839 Bentley Pl, Cerritos, CA 90703, USA;
Tel: +1.562.802.0035, Fax: +1.562.802.0625
Genernal: info@qualiherb.com

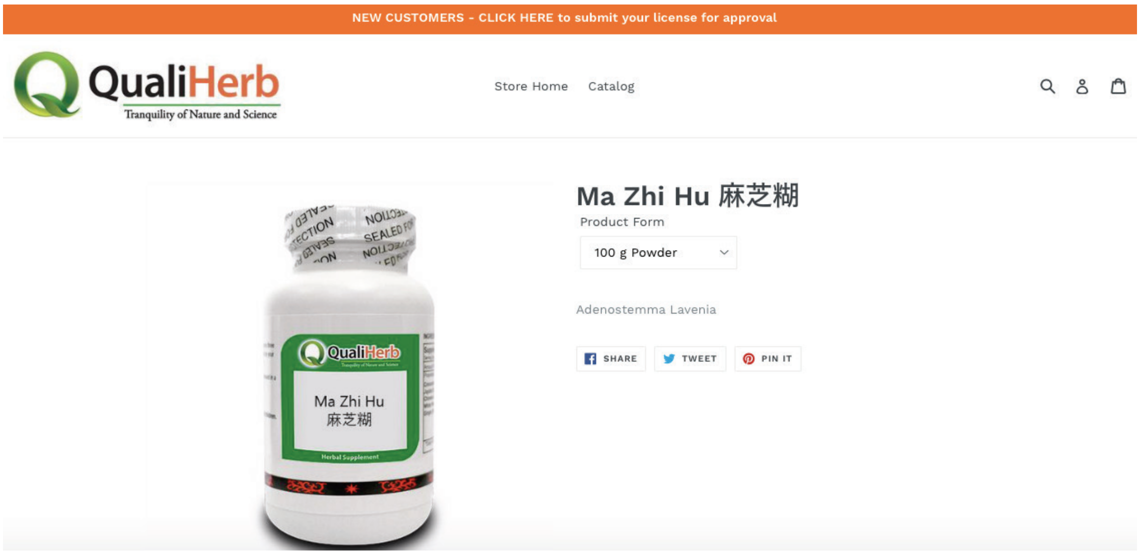

The “Ma Zi Hu” powder contains 11 α OH-KA (arrow) at a low amount, probably due to the portions of the plant, *A. lavenia,* used (the whole plant, not restricted to the leaves).

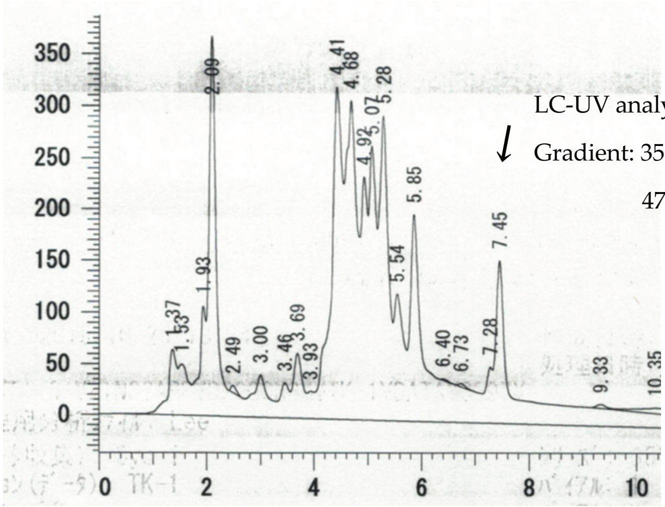

LC-UV analysis (Water extract of “Ma Zi Hu”)
Gradient: 35% - 47.5% acetonitrile/water, 10 min
47.5% - 100% acetonitrile/water, 2 min

All authors have no conflicts of interest with regard to the company QualiHerb.

Supplementary Figure 2

^{1}H-NMR spectrum of authentic and purified 11 α OH-KA.

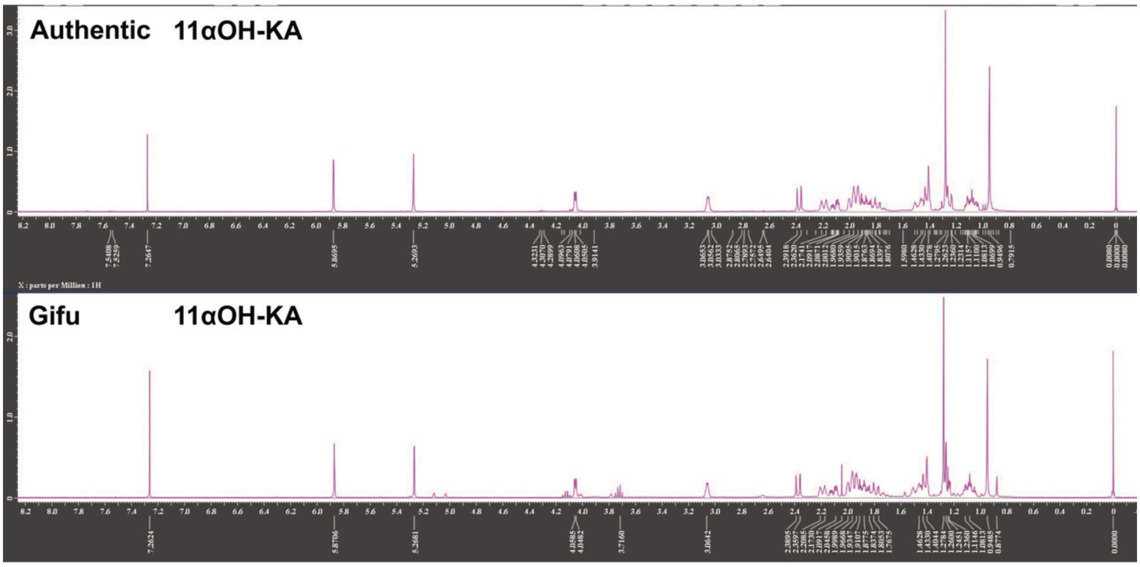

Supplementary Figure 3

LC-MS analyses of 11α OH-KA.

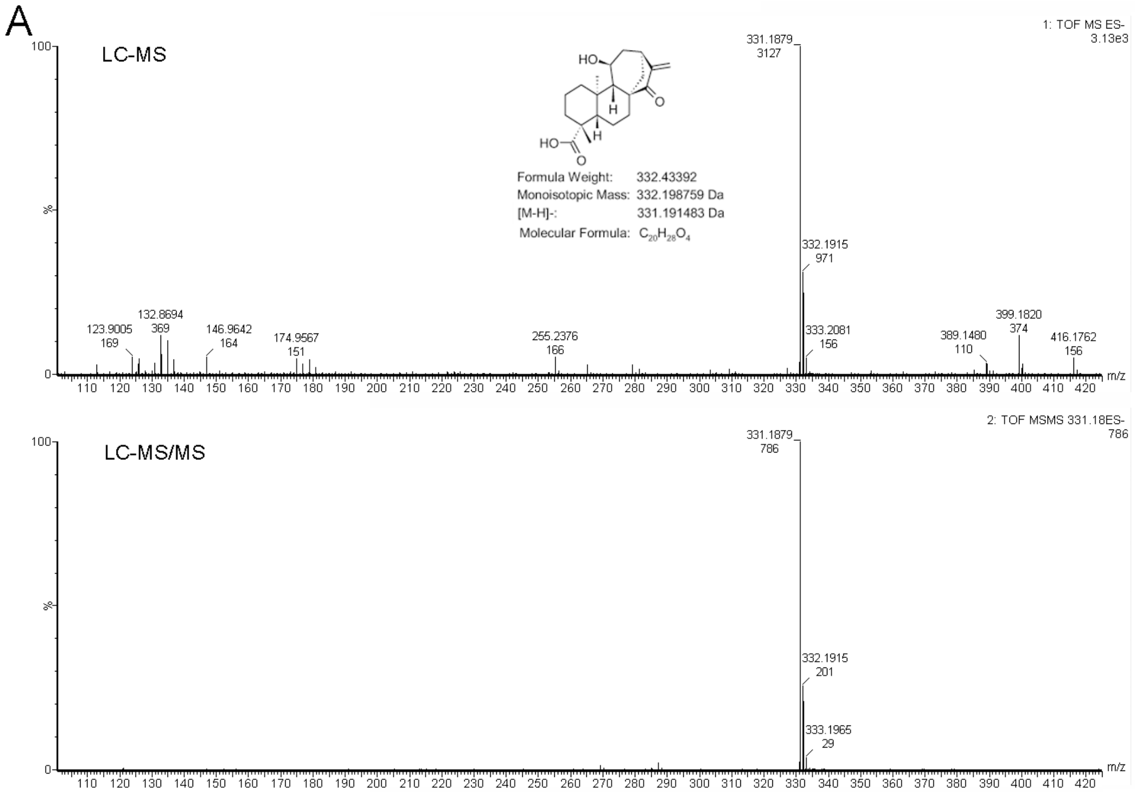

A. LC-MS analysis (*upper*) and LC-MS/MS analysis (*lower*) of authentic 11α OH-KA.

B

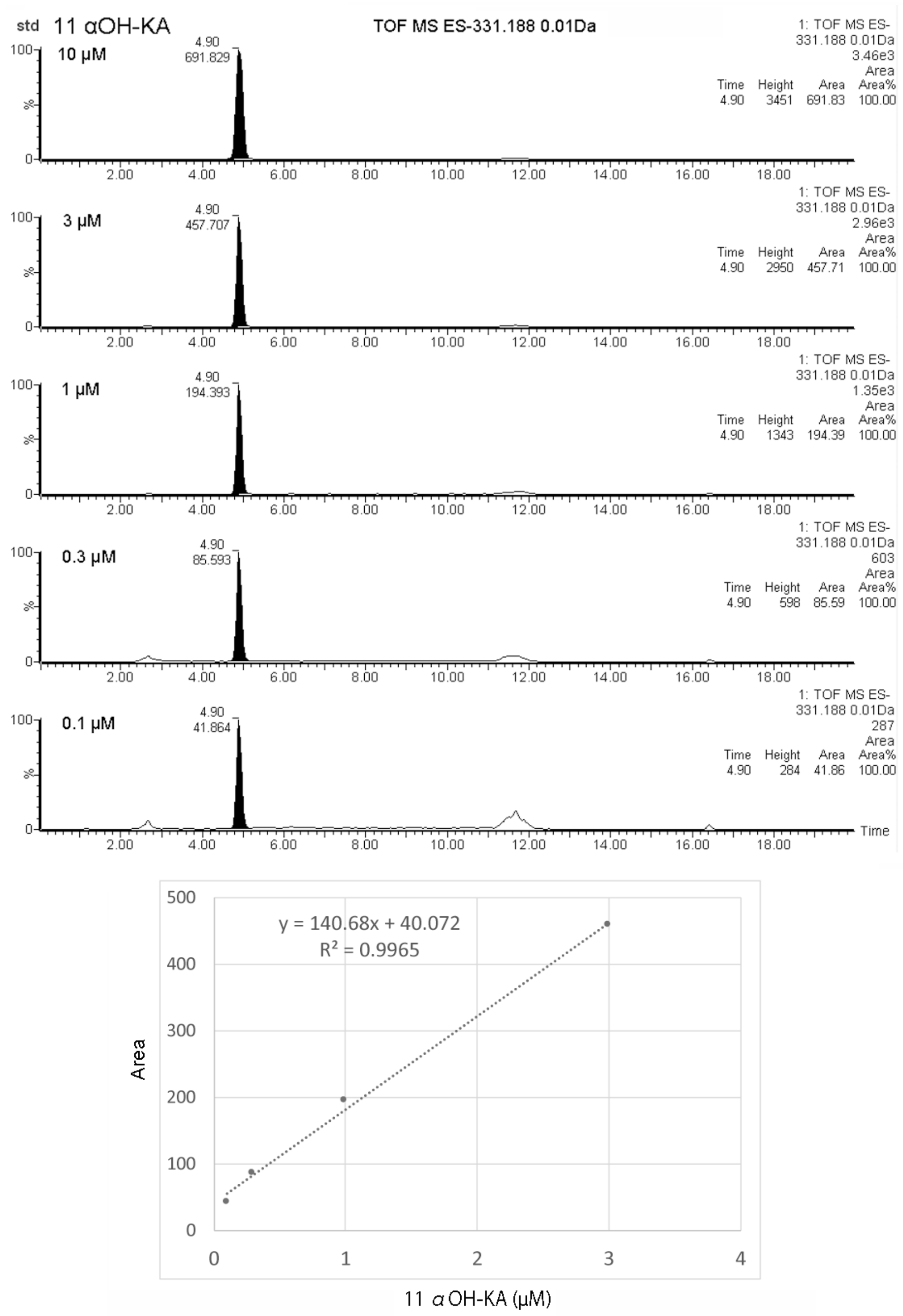

B. A standard curve of authentic 11 α OH-KA (TOF MS ES-331.188). Control mouse serum was treated (extracted) with acetonitrile according to the procedures to extract kaurenoic acid. After removal acetonitrile/water by evaporation, 50% methanol was added into tubes, and the resultant solution was used to dissolve authentic 11 α OH-KA for LC-MS analyses.

Supplementary Figure 4

An interim report of a chronic toxicity assay of *A. lavenia* leaf water extract. Four-week-old mice, C57BL/6J, freely took the water extract served as drinking water. After two months, four out of six mice had hair with suppressed pigmentation, suggesting an unknown threshold for hair pigmentation. In the future, we have to examine which is important, timing (age) or daily dose of the treatment, for the suppression of hair pigmentation. A lower mouse on a cup is an image, not related to this study.

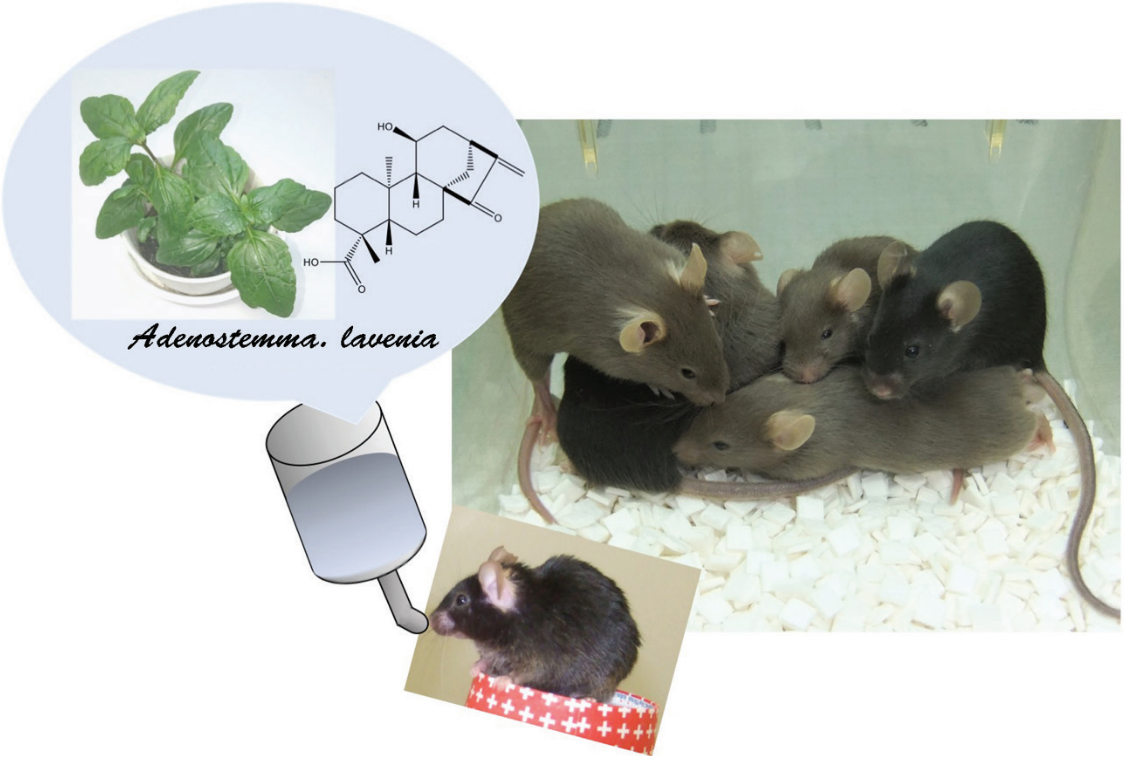

Supplement: Supplementary file 1 [file foods-09-00073-s001.pdf]
